# The current state of anesthesia safety in a third world country: a cross-sectional survey among anesthesia providers in Ethiopia

**DOI:** 10.1186/s13037-021-00290-w

**Published:** 2021-04-21

**Authors:** Fassil Mihretu

**Affiliations:** Department of Anesthesia, College of Medicine and Health Sciences, Wollo University, Dessie, Ethiopia

**Keywords:** Anesthesia, Ethiopia, Safety, Third world

## Abstract

**Background:**

Improving patient safety during anesthesia and surgery becomes a major global public health issue due to the increasing in surgical burden. Anesthesia is delivered safely in developed countries, but its safety is hampered by complex problems in third world countries. This survey assesses the unmet anesthesia needs of one of a third world country, Ethiopia.

**Methods:**

A cross-sectional survey was conducted in Amhara region of Ethiopia from 15/12/2019 to 30/1/2020. All 81 hospitals of the region were stratified by their level as district, general, and referral hospital. The study was conducted in 66 hospitals. The number of hospitals from each strata were calculated by proportional sampling technique resulting; five referral, three general, and fifty eight primary hospitals. Each hospital from each strata was selected by convenience. Each anesthesia provider for the survey was selected randomly from each hospital and questionnaires were distributed. The minimum expected safe anesthesia requirements were taken from World Health Organization-World Federation of Societies of Anesthesiologists International Standard and Ethiopian Hospitals Standard. Anesthesia practice was expected safe if the minimum requirements were practiced always (100%) in each hospital. *P* < 0.05 with 95% confidence interval were used to compare the safety of anesthesia between higher and lower level hospitals.

**Results:**

Seventy eight (88.6%) anesthesia providers working in 62 hospitals responded to the survey. On aggregate, 36 (58%) hospitals from the total 62 hospitals have met the minimum expected safe anesthesia requirements. Among the different variables assessed; professional aspects 32 (52.45%), medication and intravenous fluid 33 (53.36%), equipment and facilities 33 (52.56%), patient monitoring 43(68.88%), and anesthesia conduct 38 (62.1%) of surveyed hospitals have met the minimum requirements. Anesthesia safety is relatively higher in higher level hospitals (general and referral) 6 (75%) when compared to district hospitals 30 (55.5%), *P* < 0.001.

**Conclusion:**

Anesthesia safety in Ethiopia appears challenged by substandard continuous medical education and continuous professional development practice, and limited availability of some essential equipment and medications. Patient monitoring and anesthesia conduct are relatively good, but World Health Organization surgical safety checklist application and postoperative pain management are very low, affecting the delivery of safe anesthesia conduct.

## Introduction

The increment of non-communicable diseases, trauma and cesarean delivery makes the surgical burden higher than the past. As a result of this, improving patient safety during anesthesia and surgery becomes a major global public health issue [1, 2]. The World Health Assembly recognizes this surgical burden in 2015 and accepted strengthening emergency and essential surgical and anesthesia care as a component of universal health coverage for the first time [3]. Anesthesia safety varies from country to country depending mainly on economic background. It is extremely safe in developed countries, whereas, it lags far behind in other parts of the world [4–7]. A study conducted by Bashford in Ethiopia [8] indicates a large proportion of respondents were not able to deliver safe general (39%), spinal (50%), pediatric (63%), and obstetric (89%) anesthesia.

Professional standards, guidelines, and protocols have been developed with the sole aim of making anesthesia safer [9]. Application of current standards of anesthesia care are thought to prevent nearly 43% of operation theatre adverse events [10]. The practice of these standards in the developed world is not disputable, but it is uncertain in developing countries due to complex problems [7, 11–13], requiring frequent assessment and reporting. The aim of this survey was to assess the current unmet anesthesia need against international and national standards in one of a third world country, Ethiopia. This survey will give information for action to Ethiopian Ministry of Health, donors, researchers, and other stakeholders regarding the current state of anesthesia safety.

## Methods

Cross sectional survey was conducted in Amhara region of Ethiopia from 15/12/2019 to 30/1/2020.The study region was selected convienently from the nine regions of Ethiopia. According to the regional health sector financing report, there were 80 public hospitals (5 referral, 2 general, and 73 district) in the region [13]. These hospitals give service for nearly 21.5 million people. Recently one additonal referral hospital was opened and two primary hospitals were changed to general hospital. The total number of hospitals in the region now are 81; six referral, four general, and 71 primary.

The sample size was calculated by using Cochran’s formula for finite single population proportion,**n = z**^**2**^**pq/w**^**2**^ and **no=** $\frac{\boldsymbol{n}}{\mathbf{1}+\left(\boldsymbol{n}-\mathbf{1}\right)/\boldsymbol{N}}$.Where, **Z** is the standard normal distribution value at a/2 (1.96 at 95% confidence interval), **p** is the proportion of respondents practicing safe anesthesia in similar study (0.4) [8], **q** is 1-p (0.6), **W** is marigin of error at 5%, **N** is total number of hospitals in the region (81), **n** is required sample size (369), and **no** is the new adjusted sample size for finite population (66). So, we took 66 hospitals from the total 81 hospitals as a sample. After stratifiying all hospitals by their level as district (primary), general, and referral, the 66 hospitals were selected by proportionat sampling tecnique resulting five referral, three general, and fifty eight primary hospitals. Each hospital from each strata was selected by convinence sampling method by assuming hospitals in the same strata are homoginious. Individual respondents or each anesthesia providers from each hospital of the strata were selected randomly for actual data collection. Then questionnaire was distributed for those anesthesia providers working in the 66 hospitals. Two investigators were participated in the data collection process.

### Outcome measurements

Questionnaire was developed from the World Federation of Societies of Anesthesiologists (WFSA) anesthesia facility assessment tool [14] and previous study questionnaire applied in Ethiopia by asking the author, Bashford [8]. The questionnaire was prepared in English and not translated to other languges. It was designed by encorporating questions about professional aspects, facilities and equipments, medications and intravenous fluids, patient monitoring, and conduct of anesthesia. These questiones have response options; always (100%), almost always (76–99%), often (51–75%), sometimes (26–50%), rarely (1–25%), and never (0%). Anesthesia practice was expected safe if the minimum safe anesthesia requirments were practiced always (100%).The minimum expected safe anesthesia requirements to compare with our result were taken from World Health Organization-World Federation of Societies of Anesthesiologists (WHO-WFSA) International Standard for a Safe Practice of Anesthesia and Ethiopian Hospitals Standard (Table 1). The requirments were described in five headings; professional aspects, facilities and equipments, medications and intravenous fluids, patient monitoring, and conduct of anesthesia.

**Table 1:** Minimum expected safe anesthesia requirements to be fulfilled in each heading^a^

Professional aspect	Facilities and equipments	Medications and intravenous fluids	Patient monitoring	Conduct of anesthesia
Bachelor of science (BSc) anesthesia professional, Continuous Professional Development (CPD) and Continuous Medical Education (CMD)	Adequate lighting, tilting operating table, supply of oxygen, Oropharyngeal airways, different size facemasks, Laryngoscope for adult and pediatrics, Endotracheal tubes for adult and pediatric, intubation aids, suction device with catheter, adult and pediatric self-inflating bags, equipment for intravenous (IV) infusions and injection, equipment for spinal anesthesia, sterile gloves, defibrillator, Stethoscope, Pulse oximetry adult and pediatric, Capnography, non-invasive blood pressure monitor for adult and pediatric, and Electrocardiogram	Ketamine, diazepam or midazolam, morphine, local anesthetic (lidocaine or bupivacaine) dextrose, normal saline or ringer’s lactate, epinephrine (adrenaline), atropine, acetaminophen, NSAID (non-steroidal anti-inflammatory drugs), and magnesium	Clinical observation, using audible signals and alarms, continuous use of pulse oximetry, intermittent non-invasive blood pressure monitoring (NIBP), and carbon dioxide detector for patients undergoing intubation	Preoperative anesthesia assessment and consent, transfer of care and delegation of care, post anesthesia care unit (PACU), record keeping, WHO safe surgery checklist application, continuous presence of anesthesia provider, and pain management

^a^Developed from WHO-WFSA International Standard for safe practice of anesthesia [11], Ethiopian Primary Hospital Requirements [15], Ethiopian General Hospital Requirements [16], and Ethiopian Comprehensive Specialized Hospital requirements [17]

### Data processing and analysis

After checkeing for completeness, accuracy, and clarity, data analaysis was conducted by using Microsoft Excel and SPSS version 20. Any questionnaire with unfilled part or incomplet answer were not used for analysis. One anesthesia providers response from each hospital was taken for analysis. For more than one respondents from one hospital, the response was avaraged and taken as one respondent response for analysis. *P* < 0.05 with 95% confidence interval in Mann-Whitney U test were used to compare the safety of anesthesia between higher and lower level hospitals. Results were described by table and graph finally.

## Result

Among 88 anesthesia providers who were working in the 66 hospitals who received the questionnaire, 78 anesthesia providers (88.63%) who were working in 62 hospitals responded appropriately. There were two respondents per hospital in 10 hospitals and three respondents per hospital in two hospitals. The remained respondents were one anesthesia provider from each hospital. Anesthesia providers from four hospitals were not responded appropriately. Eight respondents were Masters holder in advanced clinical anesthesia (MSc), 68 respondents were BSc in anesthesia, and two respondents were an advanced diploma anesthesia providers. The average response was taken for more than one respondents from similar hospital (i.e. the average was described as one respondents response in the results below).

### Professional aspects

There were 206 anesthesia providers in 62 hospitals of the region one Anesthesiologist, 37 MSc Anesthesia Professionals, 153 BSc Anesthetists, and 15 Diploma Anesthesia Providers (Table 2). Among the surveyed hospitals, 60 (96.8%) had BSc Anesthetists and 4 (6.5%) had MSc Anesthesia Professionals always. Anesthesiologists were not available in any of the surveyed hospitals always, but one Anesthesiologist often available in one hospital. Only five hospitals (8.1%) gave continuous professional development and continuous medical education to their staffs always (Table 3).

**Table 2:** Number of anesthesia providers in the surveyed hospitals with description

Anesthesia provider^a^	Number	Description of anesthesia providers^b^
Anesthesiologists	1	A graduate of health science college/institute who is a Medical doctor and completed a nationally recognized specialist anesthesia training program
MSc Anesthesia Professionals	37	A graduate of health science college/ institute who have a BSc degree in anesthesia and completed a nationally recognized MSc anesthesia training program
BSc Anesthetist	153	A graduate of health science college/institute who has completed a nationally recognized BSc anesthesia training program
Advanced Diploma Anesthetist	15	A graduate of health science college/institute who has completed a nationally or regionally recognized advanced diploma anesthesia training program
Total	206	

^a^**Anesthesia provider:** Any healthcare worker who provides anesthesia care, irrespective of professional background or moderate or deep training

^b^Description was taken from WHO-WFSA International Standards and Ethiopian Hospital Standards [11, 15, 16, 18]

**Table 3:** Availability of anesthesia providers and their opportunity for continuous professional development and continuous medical education

*N* = 62	Always	Almost always	Often	Sometimes	Rarely	Never
	(100%)	(76–99%)	(51–75%)	(26–50%)	(1–25%)	(0%)
Anesthesiologist	0 (0%)	0 (0%)	1 (1.6%)	0 (0%)	1 (1.6%)	60 (96.8%)
MSc anesthesia professionals	4 (6.5%)	3 (4.8%)	1 (1.6%)	0 (0%)	0 (0%)	54 (87.1%)
BSc anesthetists	60 (96.8%)	2 (3.2%)	0 (0%)	0 (0%)	0 (0%)	0 (0%)
Diploma anesthetist	12 (19.4%)	7 (11.3%)	2 (3.2%)	4 (6.5%)	1 (1.6%)	36 (58%)
Opportunity for CPD and CMD	5 (8.1%)	2 (3.2%)	11 (17.7%)	14 (22.6%)	12 (19.4%)	18 (29%)

### Standards for medications and intravenous fluids

From the highly recommended drugs that should be present in any hospital; ketamine and atropine were the most available drugs, found in 54 (87%) of surveyed hospitals always. Morphine, dextrose, and magnesium were never accessed in 12 (19.4%), 5 (8.1%), and 12 (19.4%) of surveyed hospitals respectively (Table 4).

**Table 4:** Availability of medications and intravenous fluids

*N* = 62	Always(100%)	Almost always	Often	Sometimes	Rarely	Never
	(100%)	(76–99%)	(51–75%)	(26–50%)	(1–25%)	(0%)
Ketamine	54 (87.1%)	8 (12.9%)	0 (0%)	0 (0%)	0 (0%)	0 (0%)
Diazepam or midazolam	23 (37.1)	33 (53.2%)	6 (9.7%)	0 (0%)	0 (0%)	0 (0%)
Morphine per oral (Po)/ Iv	0 (0%)	17 (27.4%)	7 (11.3%)	19 (30.6%)	7 (11.3%)	12 (19.4%)
Local anesthetic	25 (40.3%)	30 (48.4%)	7 (11.3%)	0 (0%)	0 (0%)	0 (0%)
Dextrose	25 (40.3%)	18 (29%)	11 (17.7%)	3 (4.8%)	0 (0%)	5 (8.1%)
Normal saline or Ringer’s lactate	52 (83.9%)	10 (16.1%)	0 (0%)	0 (0%)	0 (0%)	0 (0%)
Epinephrine (adrenaline)	41 (66.1%)	14 (22.6%)	2 (3.2%)	2 (3.2%)	3 (4.8%)	0 (0%)
Atropine	54 (87.1%)	8 (12.9%)	0 (0%)	0 (0%)	0 (0%)	0 (0%)
Paracitamol Po	34 (54.8%)	25 (40.3%)	3 (4.8%)	0 (0%)	0 (0%)	0 (0%)
NSAID	40 (64.5%)	16 (25.8%)	3 (4.8%)	3 (4.8%)	0 (0%)	0 (0%)
Magnesium	16 (25.8%)	18 (29%)	5 (8.1%)	8 (12.9%)	3 (4.8%)	12 (19.4%)

### Facilities and equipments

Supply of oxygen was available in 36 (57.3%), Facemasks in 46 (74.2%), Laryngoscope (Adult and pediatric) in 35 (56.5%), and Endotracheal tubes adult and pediatric in 33 (53.2%) hospitals always. Defibrillator, intubation aids and Capnography were the list available equipments found in 0 (0%), 11 (17.7%), and 13 (21%) of surveyed hospitals always (Table 5).

**Table 5:** Availability of facilities and equipments

*N* = 62	Always	Almost always	Often	Sometimes	Rarely	Never
	(100%)	(76–99%)	(51–75%)	(26–50%)	(1–25%)	(0%)
Adequate lighting	18 (29%)	30 (48.4%)	11 (17.7%)	3 (4.8%)	0 (0%)	0 (0%)
Tilting operating table	42 (67.7%)	13 (21%)	7 (11.3%)	0 (0%)	0 (0%)	0 (0%)
Oropharyngeal airways (all size)	29 (46.8%)	26 (41.9%)	3 (4.8%)	4 (6.5%)	0 (0%)	0 (0%)
Supply of oxygen	36 (57.3%)	22 (36.2%)	4 (6.5%)	0 (0%)	0 (0%)	0 (0%)
Facemasks (all size)	46 (74.2%)	14 (22.6%)	2 (3.2%)	0 (0%)	0 (0%)	0 (0%)
Laryngoscope (for all age)	35 (56.5%)	24 (38.7%)	3 (4.8%)	0 (0%)	0 (0%)	0 (0%)
Endotracheal tubes (for all age)	33 (53.2%)	21 (33.9%)	8 (12.9%)	0 (0%)	0 (0%)	0 (0%)
Intubation aids	11 (17.7%)	19 (30.6%)	26 (41.9%)	3 (4.8%)	3 (4.8%)	0 (0%)
Suction device	30 (48.4%)	26 (41.9%)	6 (9.7%)	0 (0%)	0(0%)	0 (0%)
Self-inflating bags	45 (72.6%)	11 (17.7%)	6 (9.7%)	0 (0%)	0(0%)	0 (0%)
Equipment for spinal anesthesia	30 (48.4%)	24 (38.7%)	4 (6.5%)	4 (6.5%)	0(0%)	0 (0%)
Sterile gloves	54 (87.1%)	8 (12.9%)	0 (0%)	0 (0%)	0(0%)	0 (0%)
IV infusions and injection equipment	24 (38.7%)	21 (33.9%)	14 (22.6%)	3 (4.8%)	0 (0%)	0 (0%)
Stethoscope	49 (79%)	10 (16.1%)	3 (4.8%)	0 (0%)	0(0%)	0 (0%)
Defibrillator	0 (0%)	1 (1.6%)	8 (12.9%)	0 (0%)	0(0%)	53 (85.5%)
Pulse oximetry	50 (80.6%)	12 (19.4%)	0 (0%)	0 (0%)	0(0%)	0 (0%)
NIBP monitor	50 (80.6%)	9 (14.5%)	3 (4.8%)	0 (0%)	0(0%)	0 (0%)
Capnography	13 (21%)	6 (9.7%)	4 (6.5%)	3 (4.8%)	1(1.6%)	35 (56.5%)
Electrocardiogram	32 (51.6%)	17 (27.4%)	13 (21%)	0 (0%)	0(0%)	0 (0%)

### Standards for monitoring

**Fig. 1:**
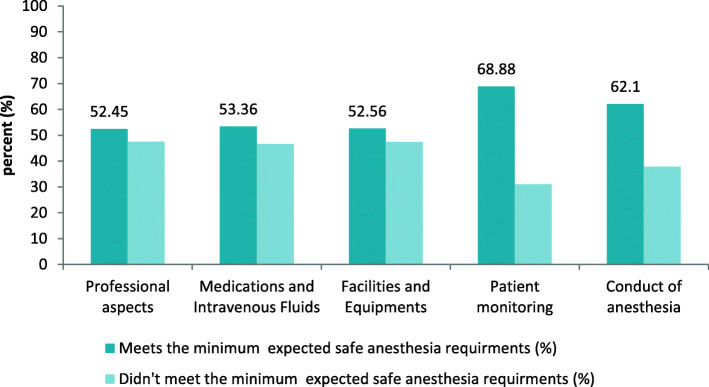
Proportion of hospitals which meets the minimum expected safe anesthesia requirements on each heading in Amhara region, Ethiopia

In average, 68.88% of the surveyed hospitals have fulfilled the minimum expected safe anesthesia requirements for patient monitoring (Fig. 1). Patients were monitored in 52 (83.9%) hospitals clinically always. Fifty seven hospitals (91.1%) used pulse oximetry continuously, 51 (82.3%) use intermittent noninvasive blood pressure monitoring (NIBP), and 47 (75.8%) use audible alarm always (Table 6).

**Table 6:** Perioperative patient monitoring

*N* = 62	Always	Almost always	Often	Sometimes	Rarely	Never
	(100%)	(76–99%)	(51–75%)	(26–50%)	(1–25%)	(0%)
Clinical observation	52 (83.9%)	10 (16.1%)	0 (0%)	0 (0%)	0 (0%)	0 (0%)
Audible signals and alarms	47 (75.8%)	15 (24.2%)	0 (0%)	0 (0%)	0 (0%)	0 (0%)
Continuous use of pulse oximetry	57 (91.1%)	5 (8.1%)	0 (0%)	0 (0%)	0 (0%)	0 (0%)
Intermittent NIBP monitoring	51 (82.3%)	11 (17.7%)	0 (0%)	0 (0%)	0 (0%)	0 (0%)
Capnography during intubation	7 (11.3%)	8 (12.9%)	8 (12.9%)	2 (3.2%)	3 (4.8%)	34 (54.8%)

### Conduct of anesthesia

Generally, on average 62.1% of the surveyed hospitals have met the minimum expected requirements for safe anesthesia conduct (Fig. 1). Preoperative assessment was done always in 47 (75.8%) of hospitals regardless of the level of anesthesia provider. World Health Organization Surgical Safety Checklist (or locally modified version) was applied in 21 (33.9%) of hospitals always. Postoperative pain was managed in 17 (27.4%) of hospitals adequately (Table 7).

**Table 7:** Conduct of anesthesia

*N* = 62	Always	Almost always	Often	Sometimes	Rarely	Never
	(100%)	(76–99%)	(51–75%)	(26–50%)	(1–25%)	(0%)
Preoperative patient evaluation	47 (75.8%)	15 (24.2%)	0 (0%)	0 (0%)	0 (0%)	0 (0%)
Use of WHO Surgical Safety Checklist	21 (33.9)	27 (43.5%)	4 (6.5%)	8 (12.9%)	2 (3.2%)	0 (0%)
Presence of anesthesia provider continuously	52 (83.9%)	7 (11.3%)	3 (4.8%)	0 (0%)	0 (0%)	0 (0%)
Documentation	46 (74.2%)	13 (21%)	3 (4.8%)	0 (0%)	0 (0%)	0 (0%)
Transfer of patients to PACU with detailed transfer of care	48 (77.4%)	7 (11.3%	4 (6.5%)	0 (0%)	0 (0%)	3 (4.8%)
Postoperative pain management	17 (27.4%)	21 (33.9%)	12 (19.4%)	6 (9.7%)	3 (4.8%)	3 (4.8%)

In addition to the result shown in each heading above, safe practice of anesthesia has significant association with level of hospitals (*p* < 0.001).Anesthesia safety was higher in higher level hospitals (general and referral) when compared to district hospitals as shown in Mann-Whitney U test result (Table 8). Anesthesia practice was safe in 6 (75%) of higher hospitals (general and referral) and 30 (55.5%) of district hospitals.

**Table 8:** Compaction between lower and higher level hospitals in meeting the minimum safe anesthesia requirements

Level of hospital	Meets minimum safe anesthesia requirement (%)	Doesn’t meets minimum safe anesthesia requirement (%)	*p*-value
District(primary) hospitals	30 (55.5%)	24 (44.44)	< 0.001
Higher level hospitals(general and referral hospitals)	6 (75%)	2 (25%)	

## Discussion

The results of this survey shows; on aggregate 58% of surveyed hospitals have met the minimum expected requirements for safe practice of anesthesia. Adequate quantities of appropriate anesthetic, analgesic, resuscitative, other adjuvant medications, intravenous fluids, equipment and facilities should be available in healthcare facilities for any patient irrespective of the patients’ ability to pay [11]. However, most low income countries including Ethiopia can’t afford this (4–8). Our result indicates, the state of safe anesthesia practice in the region was comparable with other low-income countries situation with a promising improvement. It is expected that, the percent of hospitals which met the minimum requirement would be increased if we had used always, sometimes, and never options only as used in many researches. However, almost always, often, and rarely options were added in our assessment tool.

On professional aspects, 60 (96.8%) of surveyed hospitals have BSc (nurse or none nurse) anesthesia professionals always whom covers most of the anesthesia activities in the region. The number of anesthesia providers had been increasing and becomes comparable with the total number of anesthesia providers that were in all regions of the country in 2014 [8]. Beyond the increasing in number of BSc anesthesia providers, there was a critical shortage of Anesthesiologists and MSc anesthesia professionals who have advanced training and experience. Actually shortage of trained anesthesia professionals in some low income countries resulted in administration of anesthesia by none trained professionals and referral of patients from district to other hospitals in the past [4]. These professionals with advanced training and experience were scattered in cities and referral hospitals. So, it is appreciated and cost effective to have at least BSc anesthetists in almost all hospitals especially in district hospitals.

Anesthesia is a high risk profession requiring updated knowledge and skills to maintain patients’ safety. Continuous professional development and continuous medical education practice was extremely low in the surveyed hospitals. There was only one anesthesia school in Ethiopia in 1996 [17], 3 institutions in 2005, and 26 institutions in 2016, producing mainly BSc anesthesia professionals [19]. Residency in anesthesiology and MSc in advanced clinical anesthesia has been given in three and six higher institutions respectively today with limited intake capacity. The limited intake capacity of higher institutions for advanced training results in professionals’ continuous medical education to be extremely low. On the other part, there is no PhD anesthesia training program in the country which limits MSc anesthesia professionals’ continuous medical education to the next career.

Previous studies in Ethiopia and other low-and middle income countries signify the shortage of equipment, facilities, essential drugs, and monitoring devices [4, 5, 20–25]. Our result was similar with other low-income countries regarding to equipment and essential medications with little appreciable improvement. For example, ketamine and atropine were available in 54 (87%) of the surveyed hospitals and adrenalin was available in 41 (66.1%) of hospitals always. This result is nearly similar with the result Bashford study in Ethiopia and Hodges et al. in Uganda [8, 23]. The availability of drugs in Hodges et al. and Bashford study looks higher than the result in this survey, but it seems it was due to the response options which are only; always, sometimes, and never. In a survey done in 22 low and middle income countries [21], 45.2% of facilities surveyed had uninterrupted access to oxygen either via cylinders or oxygen concentrators comparatively lower than this surveys result. Pulse oximetry was either unavailable or was only patchily available in many low-and middle-income countries including Ethiopia in the past [25, 26]. The result of this study shows, 50 (80%) of the surveyed hospitals have pulse oximetry always, indicating the improvement of anesthesia practice in some requirements. However, equipments which are very important for difficult airway management were very scarce requiring much investment because difficult airway is the major cause of anesthesia related mortality [27].

The deficiency of equipment and facilities will affect patient monitoring in turn, which is one of the greatest barriers to access safe anesthesia. Evidences suggest that patient monitoring by clinical observation, clinical examination, and using a combination of essential monitoring device increases perioperative patient outcome and safety [20, 28, 29]. Clinical observation encompasses measuring pulse rate and quality, seeing tissue oxygenation and perfusion, measuring respiratory rate and quality, observing breathing system bag movement, hearing breath sounds, hearing heart sounds and assessing pain level. It is noted that patients were monitored by clinical observation always in 83.9% surveyed hospitals. Continuous use of pulse oximetry and intermittent noninvasive blood pressure monitoring was relatively very good in the in the surveyed hospitals. In contrast, the application and availability of carbon dioxide detectors for patients undergoing intubation was extremely low.

The general conduct of anesthesia was relatively good with the deficiency of equipments, facilities, drugs, and continuous professional development practice. However, there was a lag behind in the practice of some standards, most specifically on the usage of WHO Surgical Safety Checklist or its modified version and postoperative pain management. It is known that application of WHO Surgical Safety Checklist decreases anesthesia and surgery-related mortality and morbidity [30–32]. A study in Felegehiwot referral hospital by Ellis, et al. [33] and Yekatit 12 hospital by Bashford et al. [34] in Ethiopia shows the application of the WHO checklist was similarly low in their baseline information. Postoperative pain management was inadequate in the surveyed hospitals and similar study in Ethiopia shows its inadequacy [35, 36]. Generally, higher level hospitals (general and referral) have practiced anesthesia more safely than district hospitals with regard to the minimum requirements (highly recommended standards). There are standards which should be fulfilled by higher level hospitals (recommended and suggested) but not by district hospitals. This survey didn’t study weather higher level hospitals were meeting these standards or not which is one of its limitations.

## Conclusion

Anesthesia safety in the surveyed hospitals is far from the minimum expected standards by some requirements like continuous professional development, continuous medical education, and application of WHO surgical safety checklist. The general progress towards the accomplishment of the safe minimum criteria was good. High investment on continuous medical education and continuous professional development and moderate investment on essential equipment, medications, and facilities is needed to attain at least the minimum safe anesthesia requirements. Future surveys are needed to fill the gaps of information to government officials, donors and other stakeholders.

## Data Availability

The data used to support the findings of this survey are available from the corresponding author upon request.

## Abbreviations

BSc: Bachelors of Science
CMD: Continuous Medical Education
CPD: Continuous Professional Development
IV: Intravenous
MSc: Masters of Science
NIBP: Noninvasive Blood Pressure
NSAID: Non-steroidal Anti-inflammatory Drugs
PACU: Post Anesthesia Care Unit
Po: Per Oral
WFSA: World Federation of Societies of Anesthesiologists
WHO: World Health Organization
